# Prognostic Impact of Array-based Genomic Profiles in Esophageal Squamous Cell Cancer

**DOI:** 10.1186/1471-2407-8-98

**Published:** 2008-04-11

**Authors:** Ana Carneiro, Anna Isinger, Anna Karlsson, Jan Johansson, Göran Jönsson, Pär-Ola Bendahl, Dan Falkenback, Britta Halvarsson, Mef Nilbert

**Affiliations:** 1 Department of Oncology, Lund University Hospital, 221 85 Lund, Sweden; 2 Department of Clinical Oncology, Portuguese Institute of Oncology, 1099-023 Lisbon, Portugal; 3 Department of Surgery, University Hospital, 221 85 Lund, Sweden; 4 Department of Surgery, Helsingborg Hospital, 251 87 Helsingborg, Sweden; 5 Department of Pathology, Helsingborg Hospital, 251 87 Helsingborg, Sweden; 6 Department of Clinical Sciences, Copenhagen University and Clinical Research Unit, Hvidovre Hospital, 2650 Hvidovre, Denmark

## Abstract

**Background:**

Esophageal squamous cell carcinoma (ESCC) is a genetically complex tumor type and a major cause of cancer related mortality. Although distinct genetic alterations have been linked to ESCC development and prognosis, the genetic alterations have not gained clinical applicability. We applied array-based comparative genomic hybridization (aCGH) to obtain a whole genome copy number profile relevant for identifying deranged pathways and clinically applicable markers.

**Methods:**

A 32 k aCGH platform was used for high resolution mapping of copy number changes in 30 stage I-IV ESCC. Potential interdependent alterations and deranged pathways were identified and copy number changes were correlated to stage, differentiation and survival.

**Results:**

Copy number alterations affected median 19% of the genome and included recurrent gains of chromosome regions 5p, 7p, 7q, 8q, 10q, 11q, 12p, 14q, 16p, 17p, 19p, 19q, and 20q and losses of 3p, 5q, 8p, 9p and 11q. High-level amplifications were observed in 30 regions and recurrently involved 7p11 (*EGFR*), 11q13 (*MYEOV, CCND1, FGF4, FGF3, PPFIA, FAD, TMEM16A, CTTS *and *SHANK2*) and 11q22 (*PDFG*). Gain of 7p22.3 predicted nodal metastases and gains of 1p36.32 and 19p13.3 independently predicted poor survival in multivariate analysis.

**Conclusion:**

aCGH profiling verified genetic complexity in ESCC and herein identified imbalances of multiple central tumorigenic pathways. Distinct gains correlate with clinicopathological variables and independently predict survival, suggesting clinical applicability of genomic profiling in ESCC.

## Background

Esophageal squamous cell carcinoma (ESCC) is a major cause of cancer-related mortality worldwide. Despite advances in diagnostic methods and combined treatment modalities, the majority of the tumors are diagnosed at advanced stages and the overall 5-year survival rate remains 40%. ESCC develops through a multistep process from dysplasia, through carcinoma *in situ *to invasive carcinoma, and the acquisition of genetic alterations is tightly related to the dysplasia-carcinoma sequence [1]. The characterization of genetic alterations inherently linked to ESCC development and an in-depth understanding of the molecular mechanisms underlying carcinogenesis and growth control may therefore provide information relevant for early tumor detection, refined prognosis and development of novel targeted therapeutics.

Loss of heterozygozity (LOH) studies and conventional comparative genomic hybridization (CGH) analyses have demonstrated genetic complexity in ESCC and have identified multiple recurrent copy number alterations, namely gains of 3q, 5p, 7q, 8q, 11q, 12p, 20p and 20q [2-6]. Amplifications of regions harbouring oncogenes e.g. 7p12 (*EGFR*), 8q24 (*MYC*), 17q21 (*FGFR*) and 11q13 (e.g. *CCND1, FGF4/3*, and *EMS1) *have consistently been observed [7-12]. Losses, albeit at a lower frequency than gains, have recurrently involved 3p, 5q, 9p, 13q, 18q and 21q and include target genes such as *FHIT, APC, RB1 *and *CDKN2A *[3,13-16]. Moreover, some of the changes identified, e.g. gain of 8q24, 11q13, 12p, and 20q12 and loss of 3p have been associated with poor prognosis, but genetic alterations and biological characteristics have so far had a limited impact on clinical prognostication and treatment. Taken together, these findings suggest that genetic profiling can be a useful diagnostic tool in ESCC, whereas its prognostic role remains uncertain. We used array-based CGH (aCGH) for high resolution mapping in 30 ESCC and demonstrate that copy number changes detected by aCGH provide prognostic information beyond that of classical clinicopathological variables.

## Methods

### Tumor tissue

All 30 patients, 24 men and 6 women with a mean age of 64 (range 54–78) years, were recruited from the southern Sweden health care region and had undergone primary esophagectomy at the Department of Surgery, Lund University Hospital. None of the patients had received neoadjuvant radiotherapy or chemotherapy. Tumor tissue collected at surgery was stored at -80°C until DNA extraction. Stage (according to the International Union Against Cancer) was I in 3 cases, II in 9, III in 7, and IV in 11 cases (see Additional file 1). Presence of = 50% tumor cells in the tissue was verified by touch imprints, which were stained with Hematoxilin-Eosin and evaluated by a gastrointestinal pathologist (B.H.). All deaths were ESCC related, the median follow up was 21 (range 1–46) months for the survivors. Written informed consent was provided by all patients and the study was approved by the Lund University ethics committee.

### BAC Array Platform

We used the 32 k human genome high-resolution BAC re-arrayed clone set, Version 1.0 from the BACPAC Resource Center at Children's Hospital Oakland Research Institute, Oakland (CA, US), produced at the Swegene DNA Microarray Resource Center (GEO platform repository accession GPL4723) [17], Department of Oncology, Lund University with a resolution >80 kb.

### DNA isolation, labelling and hybridization

DNA was extracted, labelled and hybridized as previously described [17]. A commercial obtained DNA, derived from a pool of normal human males (Promega, Madison, WI, USA) was used as reference. Scanning was performed using an Agilent microarray scanner (Agilent Technologies, Palo Alto, CA, USA).

### Image processing and data analysis

Scanned arrays were analyzed using Gene Pix Pro 4.1 (Axon Instruments, MDS Analytical Technologies, Ontario, Canada) and bad spots were "flagged" during manual inspection. The quantified data matrix was loaded into Bio Array Software Environment (BASE)[18] and filtering and normalization were performed herein [19]. Correction of background intensities of Cy3 and Cy5 were calculated using median-feature and median-local background intensities of the uploaded file. Intensity ratios were calculated from the background tumor channel (ch1) divided with the reference channel (ch2). Spots flagged as bad were filtered out from further analysis and were regarded as missing values. A signal to noise ratio (SNR) = 5 was set for both channels. Data were normalized using an implementation of a pin-based Lowess algorithm [20] in BASE excluding the X chromosome. A moving average smoothing algorithm with a 200 kbp sliding window was used and a BASE-adapted CGH-plotter software was used to identify regions of gains and losses excluding the X and the Y chromosomes [21]. A region of gain or loss was defined as two or more consecutive clones showing an absolute log_2 _ratio ≥ 0.2 and high-level amplifications as a log_2 _ratio ≥ 1.5.

### Statistical Analysis

A Chi2 test was used to identify differences between copy number changes and clinicopathological characteristics. For survival analysis, the Kaplan Meier method was used to estimate relevant event variables, and the log-rank test was used to compare survival between two strata. The Cox proportional hazards model was used for univariate and multivariate survival analyses. Cox analysis was first used for all clones, thereafter two or more consecutive significant (*P *< 0.05) clones (interrupted only by one or two clones) were re-analysed as regions affected by gains and losses. Regions significantly (*P *< 0.05) linked to stage/outcome in univariate analysis were included in the multivariate analysis. All covariates were evaluated for adherence to the assumption of proportional hazards by calculating Schoenfeld residuals. Correlations between changes within a same pathway were analysed using pairwise correlation. CGH profiles were classified as gains or losses according to the dominating variable in the region corresponding to the gene locus. After Bonferroni correction correlations with *P *< 0.05 were considered significant. Stata 9.2 (StataCorp LP, College Station, TX 77845, USA) was used for the statistical calculations.

### Immunohistochemical Staining

Serial 4-μm sections from one representative paraffin-embedded tumor block were used for immunostaining using a monoclonal antibody against human EGFR at a dilution of 1:50 (DAKO, Glostrup, Denmark). The slides were evaluated as no staining; 1+ (cytoplasmic staining or discontinuous membrane staining); 2+ (membrane staining with moderate intensity), and 3+ (intense staining with retained membranous staining). Interpretation of the staining was performed by two of the authors (AC and MN), who were blinded to the copy number changes and the clinical data.

## Results

The median number of losses per tumor was 6.9% clones (range 0.1–24%) and the median number of gained clones was 9.8% (range 1.1–21%). Recurrent gains identified in at least 60% of the tumors involved chromosome regions 5p, 7p, 7q, 8q, 10q, 11q, 12p, 14q, 16p, 17p, 19p, 19q, and 20q. Likely target genes include *TERT*, *EGFR*, *MYC*, *MYEOV*, *CCND1*, *FGF4, FGF3*, *CTTN *and *AKT1*. Loss of genetic material in at least 40% of the samples affected 3p, 5q, 8p, 9p and 11q (Table 1). A homozygous deletion of 9p21.3, corresponding to the *CDKN2A *locus, was identified in one case and was verified with PCR using *CDKN2A*-specific primers (sequences available from the authors upon request, data not shown). High-level amplifications were observed in 33 regions and recurrently involved 11q22 (harbouring *PDFG *in 3 tumors), 11q13 (in 11 tumors, encompassing *MYEOV, CCND1, FGF4, FGF3, PPFIA, FAD, TMEM16A, CTTS *and *SHANK2*) and 7p11 (including *EGFR *in 4 tumors). Overexpression of EGFR was validated by immunohistochemistry, which revaled a highly positive (3+) staining in 19 tumors, 12 of which had copy number gain of 7p11. All 4 tumors with HLA of the *EGFR *locus showed 2+ or 3+ EGFR staining.

**Table 1:** Summary of the most frequent copy number gains and losses. Table listing the most frequent copy number gains and losses, sorted in decreasing order, in relation to tumor stage. Columns give the cytoband, start reporter, end reporter, size (Mbp), and candidate genes in the region.

**Cytoband**	**Start reporter**	**End reporter**	**Size (Mbp)**	**Candidate genes**
**Gains (≥ 60%)**				

**5p15.33**	RP11-811I15	CTD-2296H22	1.8	*TERT*
**7p22.3**	CTD-2245C5	RP11-745M18	2.3	*MAD1L1, NUDT1*
**7p11.2**	RP11-449G3	RP11-535N12	1.1	
**8q24.13-q24.23**	RP11-150N13	RP11-141J23	10.5	*MYC, WISP1*
**8q24.3**	CTD-2330C15	CTD-2300I18	1.5	*FOXH1*
**10q26.3**	RP13-137A17	RP11-1065F16	0.8	
**11q13.3-11q13.4**	RP11-433A18	RP11-574F24	1.8	
**12p13.33**	CTD-2094C14	RP11-574G8	0.2	
**14q32.33**	CTD-2344P12	RP11-603L1	0.4	*AKT1*
**16p13.3**	RP11-344L6	RP11-680M24	3.2	
**17p13.1**	RP11-205D17	RP11-63C7	0.6	
**19p13.3**	RP11-519F9	RP11-81M8	1.6	
**19q13.42**	CTD-2503B7	RP11-528E9	0.3	
**20q13.33**	CTD-2022N11	RP11-350G15	2.4	

**Losses (≥ 40%)**				

**3p26.3-p24.2**	RP11-359E9	RP11-512O18	3.5	RARB, TOP2B
**3p14.2-p14.1**	RP11-350E21	RP11-175F9	6.9	*FHIT*
**3p14.1-p13**	RP11-607B7	RP11-744B4	5.8	
**5q12.3-q13.1**	RP11-158G8	RP11-633F2	0.9	*PIK3R1*
**8p23.2**	RP11-46M15	RP11-593J22	2.2	
**9p24.3-p24.2**	RP11-272N16	RP11-778P24	3.1	*MTAP, CDKN2A, CDKN2B*
**9p23**	RP11-6H18	RP11-312G1	1.9	
**9p21.3**	RP11-113D19	RP11-16P12	5.2	
**11q25**	RP11-217L21	CTD-2270L17	2.4	*ATM*

To evaluate whether genes from a same signaling pathway were correlated, a complementary analysis of pairs of genomic loci harboring genes (*CDKN2A*, *MDM2*, *RB*, *MDM4*, *CDKN1A*, *PIK3CA*, *PTEN*, *AKT1*, *TP53*, *MYC*, *CCND1*, *CCNE1*, *BCL2*, *CDK4*, *E2F3*) from central tumorigenic pathways was performed. Significant correlations (*P *< 0.05) were identified between gain of the *MDM4 *locus and loss of the *RB *locus, gain of *MDM2 *and gain of *TP53*, gain of *BCL2 *and loss of *CDKN1A*, gain of *BCL2 *and loss of *CDKN2A*, gain of *CCND1 *and loss of *CDKN2A*. Significant negative correlations were found between gain of *MDM2 *and gain of *PIK3CA*, gain of *PIK3CA *and loss of *E2F3*, loss of *PTEN *and gain *AKT1*, and concordant gain of *AKT1 *and gain of *CDK4*. Pairwise correlation analysis between members of the EGFR pathway identified significant (*P *< 0.05) correlations between gains of *AKT1/HER3*, *MAPK1/PIK3CA*, and *MAPK1/AKT1 *and between gain of *EGFR *and loss of *PTEN*.

**Figure 1:**
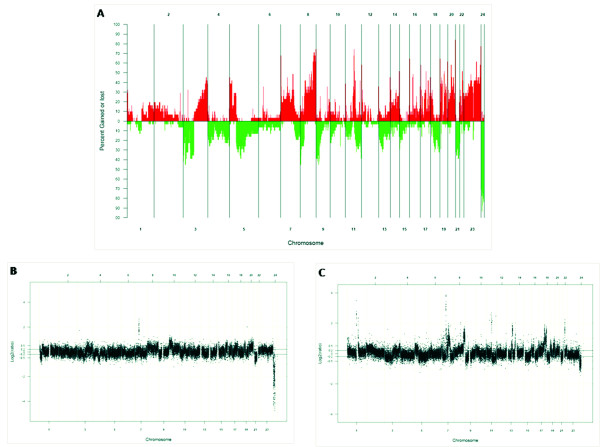
***A) *Genome-wide frequency plot of DNA copy number gains (red) and losses (green) for all 30 ESCC tumors. *B) ***Genome-wide copy number profile for a Stage I tumor (ESCC 33). Characteristic alterations for a Stage I tumors are gains on 6p, 12p, and 17p and losses 21q. ***C) ***Genome-wide copy number profile for a Stage IV tumor (ESCC 51). Characteristic for Stage IV tumors are gains on 12p, 19q and 20q and frequent losses on 3p, 5q and 9p.

Despite genetic complexity (Figure 1), the genomic profiles were found to correlate with tumor stage, differentiation, and development of metastases with a lower (mean 13% *versus *25%) number of changes in highly differentiated ESCC than in poorly differentiated tumors (see Additional file 2). A Chi2 test identified ~400 clones that mapped to 6 genomic regions (2p11.1-2q11.2, 2q35, 3p21.31, 4q12, 4q21.3-4q28.3, 5q12.1) that were significantly (P < 0.05) more often found in poorly differentiated tumors. The chromosomal regions most frequently affected by copy number gains differed between stage I and stage II-IV tumors. Gains of 6p25.3, 12p13.33 and 17p13.1 were present in all stage I tumors. Stage II-IV tumors showed a higher number of changes, with the most common gains affecting 12p13.33 (100%), 5p15.33 (85%), 20q13.33 (85%) and 11q13.3 (77%). Overall, copy number losses were more common in stage II tumors and the most frequent losses were shared by different tumor stages without a significant difference in frequency (see Additional file 3 and 4).

Smaller (pT1) tumors had the lowest number of copy number changes (mean 12% of the clones affected), whereas pT2-T4 tumors showed alterations of 27%, 16%, and 18% of the clones. When gains/losses were correlated to presence of lymph node metastases, 838 clones that mapped to 21 genomic regions were identified (see Additional file 5). Copy number gain in 7p22.3 was significantly (*P *= 0.01) associated with lymph node metastases, and correctly predicted nodal metastases in 63% of the patients, as represented by an area under ROC curve of 0.73. Changes in 19 regions, corresponding to 1074 clones, correlated to metastasis at diagnosis (see Additional file 6) with copy number gain of 8q21.3 being significantly associated with the presence of metastasis (*P *= 0.03). Gain of 8q21.3 could classify 60% of the patients with distant metastasis with 40% sensitivity and 70% specificity.

**Figure 2:**
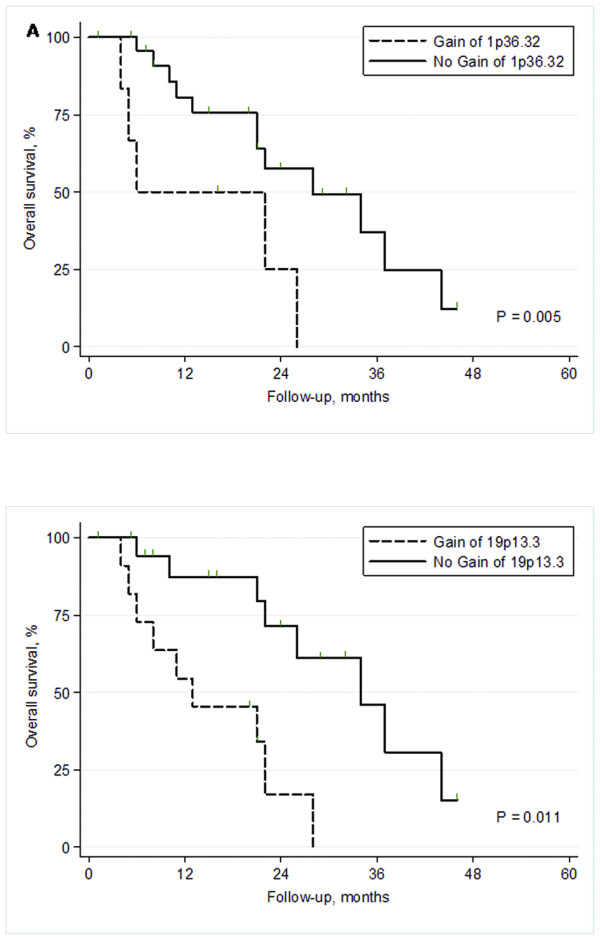
**Kaplan-Meier survival plots of the two prognostic regions in multivariate analysis.* A) ***Highly significant difference in survival between patients without gain and with gain of 1p36.32 (*P *= 0.005). ***B) ***Difference in survival between patients without gain and with gain of 19p13.3 (*P *= 0.01).

Univariate analysis verified that stage and tumor size were associated with prognosis; stage HR 1.6, *P *= 0.04 and pT HR 1.8, *P *= 0.05. When copy number gains and losses were correlated to prognosis, Cox proportional hazards analysis identified 1284 clones, with a p-value <0.05, mapping to 30 regions (see Additional file 7). When gains and losses were separately considered, 7 regions remained significantly associated to prognosis in univariate analysis. When these regions were entered with stage into multivariate analysis, gain of 1p36.32 and gain of 19p13.3 independently predicted poor prognosis (Table 2 and Figure 2).

**Table 2:** Regions correlated to prognosis in univariate and multivariate Cox analysis. Table listing the regions significantly correlated to prognosis in univariate and multivariate analysis. Columns give the type of aberration, cytoband, hazard ratio (HR), P-value and confidence interval (CI).

**A – Univariate Cox analysis**				
**Aberration**	**Cytoband**	**HR**	***P***	**CI**

Gain	19p13.3	5.0	0.005	1.6 – 15.5
Gain	3q11.2	6.4	0.012	1.5 – 26.8
Loss	10p11.23	10.3	0.013	1.6 – 65.4
Loss	9q34.3	27.5	0.019	1.7 – 439
Gain	3q22.3	3.7	0.025	1.1 – 11.6
Loss	10q11.21	6.2	0.026	1.2 – 31.1
Gain	1p36.32	3.4	0.030	1.1 – 10.6

**B – Multivariate Cox analysis**				

**Aberration**	**Cytoband**	**HR**	***P***	**CI**

Gain	1p36.32	19.6	0.005	2.5 – 153.9
Gain	19p13.3	7.0	0.011	1.5 – 31.9
Stage		2.0	0.077	0.9 – 4.4

## Discussion

Array-based genomic profiling of ESCC confirms the genetic complexity suggested by earlier studies that have applied other means of genetic profiing, e.g. cytogenetics, conventional CGH, and LOH analysis. We found copy number gains and losses affecting median 19% of the genome, identified multiple high-level amplications, and demonstrated an association between copy number alterations and stage, differentiation and prognosis, suggesting clinical applicability of genomic profiling in ESCC.

The 11q13 region is central in ESCC development and alterations herein were identified in 20/31 tumors. *CCND1 *is a likely target, but several other candidate genes, e.g. *FGF4*, *FGF3*, *CTTN *and *SHANK2*, showed high-level amplification. This amplicon harboured *MYEOV*, which has previously been associated with ESCC and described to be co-amplified with *CCND1 *[22]. The RB pathway is frequently targeted in ESCC carcinogenesis [23-25] and its activation seems to be dependent mainly on *CCND1 *amplification. In our sample set no significant correlations were observed between the gains/losses observed in the members of the *RB *pathway (*CCND1, CCNE1, E2F3 *and *CDKN2A*). Gain of 14q32.3, which includes the *AKT1 *oncogene, was identified in half of the samples. The *PTEN*-*PIK3CA*-*AKT *signalling cascade is frequently deregulated in several types of cancers and expression of *PIK3CA *has been strongly associated with elevated AKT activity. An increased copy number of *PIK3CA *is primarily detected in tumors with retained PTEN expression [26], and indeed, none of the 11 tumors with *PIK3CA *gain showed loss of the *PTEN *locus at 10q23.3, whereas 7 tumors showed *PTEN *loss without change at *PIK3CA *locus. The pairwise analysis showed a negative (*P *= 0.005) correlation of both copy number gains. Expression data from array-based oligonucleotide arrays were available from 8 samples (unpublished data) and verified overexpression of *PIK3CA *in 7 of these tumors, which further supports *PIK3CA *and *PTEN *acting as mutually exclusive tumorigenic events [27]. Gain of 7p11.2 was identified in half of the tumors and included high-level amplifications in 4 tumors. The most likely target gene herein, *EGFR*, is overexpressed in a multitude of malignancies and including ESCC [7,28-30]. Immunostaining for EGFR was highly positive (3+) in 12 out of 14 tumors with copy number gain of *EGFR*, thus suggesting, as previously reported [28,31,32], that copy number gain leads to high protein expression in a significant fraction of tumors (86% in our sample set).

In our cohort, loss of *PTEN *was observed in 23% of the samples and was significantly correlated (*P *= 0.04) to *EGFR *gain, which may be relevant for resistance to EGFR inhibitors, since *PTEN *loss correlates with treatment resistency. Furthermore, gain of 17q12, harbouring *ERBB2*, was observed in 9 tumors and 6 of these showed concomitant gain of 7p11.2 (*EGFR*), which suggests that co-overexpression of ERBB2 and EGFR may apply also to ESCC [33]. High level amplification of *ERBB2 *correlated to overexpression (data not shown). Copy number gain of 5p15 was among the most frequent changes and the minimal region of overlap harbour some 20 identified genes, among which the telomerase regulator *TERT*, which has previously been shown to be overexpressed in ESCC and has been associated with prognosis in other tumor types [12,34-36]. Gains of 7p22.3, 8q22.3-qter and 20q11.21 were also frequently found and include the target genes *MAD1L1 involved in TERT *transcription, *LRP12 *and *WISP1 *linked to cell survival and p53-mediated apoptosis and *TPX2 *known to activate Aurora-A kinase [37-41]. High-level amplifications affected 33 loci, among which recurrent high-level amplification peaks were detected at 7p11 (*EGFR)*, 11q22 (*cIAP1*, *MMP3 *and *PDGF)*, 11q13 (that harbours e.g. *CCND1, FGF4*, *FGF3*, *CTTN *and *SHANK2)*, and 10q21 with unknown targets.

The most frequent recurrent copy number losses affected 3p, 5p, 8p, 9p, and 11q, which is consistent with other studies and these loci also contain several tumor suppressors linked to ESCC [2,3,8,42]. Losses affecting the 9p21-p24 region, which contains *CDKN2A *and *CDKN2B*, were identified in 13/30 tumors. *CDKN2A *deletions have been associated with an invasive and metastatic phenotype and a homozygous *CDKN2A *deletion was identified in one sample [43-45]. Frequent losses were also observed at 3p26-p14 which harbours *THRB*, *RARB*, *TOP2B *and *FHIT*. Pairwise correlations between frequently observed gains and losses identified 5 regions that were significantly more often affected by concurrent aberrations. Four of these were located on the same chromosome, whereas loss of 3p24 and 5q12 occurred at an increased incidence. These regions contain targets such as *TOP2B*, *RARB*, and *TGFBR2 *on 3p and *PIK3R1 *and *RAD17 *on 5q, and the association identified may indicate cross-talk between genes in these regions.

## Conclusion

The accumulation of genetic changes is central in ESCC development and progression. Our study is the first to apply high-resolution aCGH to clinical prognostication in ESCC. Studies that have applied traditional CGH have suggested a prognostic independent role for genes located on 8q, 11q, 12p, 14q, and 20q [2,16,46], and a recent study using a 4k BAC array platform (with 1 Mb resolution) identified 4 clones, mapping to 3q29, 4q21.21, 8q24 and 8q24.3, linked to survival [47]. The high-resolution data here presented demonstrates extensive genetic complexity already in early stage tumors, supports the involvement of several key genes in ESCC, links gain of 7p22.3 to presence of nodal metastases and demonstrates that gains of 1p36.32 and 19p13.3 provide independent prognostic information (HR = 19.6 and 7.0 respectively). The different prognostic regions identified may be related to the inherent genetic complexity of ESCC, to differences in materials (e.g. study populations from different geographic areas with disparities in dietary and environmental exposures) and use of different genetic profiling technologies [48,49]. Nevertheless, these results hold promise for the application as genetic classifiers and refined prognostic markers. Moreover, the recognition of recurrent rearrangements in central signaling pathways provides a basis for the development of selected and individualized targeted therapeutics in ESCC.

## Competing interests

The author(s) declare that they have no competing interests.

## Authors' contributions

AC designed the study, performed experiments, analyzed the data and drafted the manuscript. AI contributed to the design of the study, performed experiments and helped to draft the manuscript. AK carried out experiments and analyzed data. JJ provided cancer samples and clinical data. GJ helped the data analysis. POB performed and supervised statistical analysis. DF provided cancer samples. BH performed pathological analysis of tumor samples. MN conceived, designed and coordinated the study, and helped to draft the manuscript. All authors read and approved the final manuscript.

## Pre-publication history

The pre-publication history for this paper can be accessed here:



## Supplementary Material

Additional file 1 Clinicopathologic variables of tumors and survival of patients of the sample set. Table listing the clinicopathologic variables of tumors and survival of patients of the sample set analyzed in this study. Click here for file

Additional file 2 Number of gains and losses by differentiation. Boxplot of the number of gained and lost clones according to tumor differentiation. Click here for file

Additional file 3 Number of gains and losses by tumor stage. Boxplot of the number of gained and lost clones according to tumor stage. Click here for file

Additional file 4 Copy number alterations, listed in decrescent prevalence order, in relation to tumor stage. Table listing the most frequent copy number alterations, sorted in decreasing order, in relation to tumor stage. Columns give the cytoband, start reporter, end reporter, size (Mbp), frequency of the change and candidate genes in the region. Click here for file

Additional file 5 Chi2 test significant regions associated with positive lymph nodes (P < 0.05). Table listing the regions of aberrations that showed a significant statistical (*P *< 0.05) association with positive lymph nodes. Given are cytoband, start reporter, end reporter, size (Mbp) and the frequency across patients with positive lymph nodes and patients with negative lymph nodes. P-values were computed using Chi2 test. Click here for file

Additional file 6 Chi2 test significant regions associated with metastasis at diagnosis (P < 0.05). Table listing the regions of aberrations that showed a significant statistical (*P *< 0.05) association with metastasis at diagnosis. Given are cytoband, start reporter, end reporter, size (Mbp) and the frequency across patients with metastasis at diagnosis and patients without metastasis at diagnosis. P-values were computed using Chi2 test. Click here for file

Additional file 7 Regions correlated to prognosis identified by Cox analysis. Table listing the regions of aberrations significantly correlated (*P *< 0.05) to prognosis in univariate Cox analysis. Cytoband, start reporter, end reporter and size (Mbp) of the aberrant region are given. Click here for file
