# Supplementary figures and images for: Prognostic Impact of Array-based Genomic Profiles in Esophageal Squamous Cell Cancer
Source: BMC Cancer. 2008 Apr 11;8:98. doi: 10.1186/1471-2407-8-98 (PMC2374796; doi:10.1186/1471-2407-8-98)

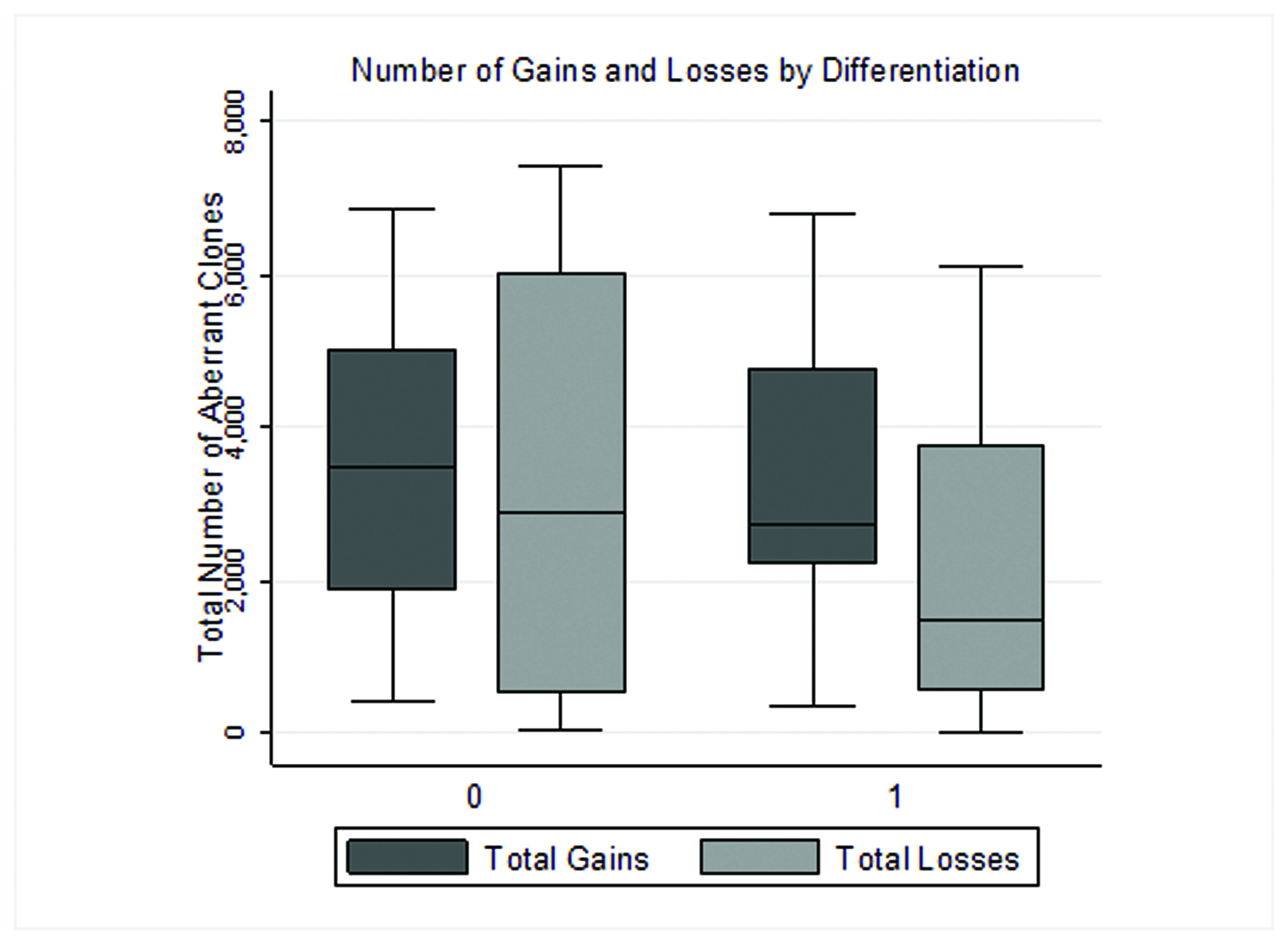

Supplement: Additional file 2 — Number of gains and losses by differentiation. Boxplot of the number of gained and lost clones according to tumor differentiation. [file 1471-2407-8-98-S2.tiff]

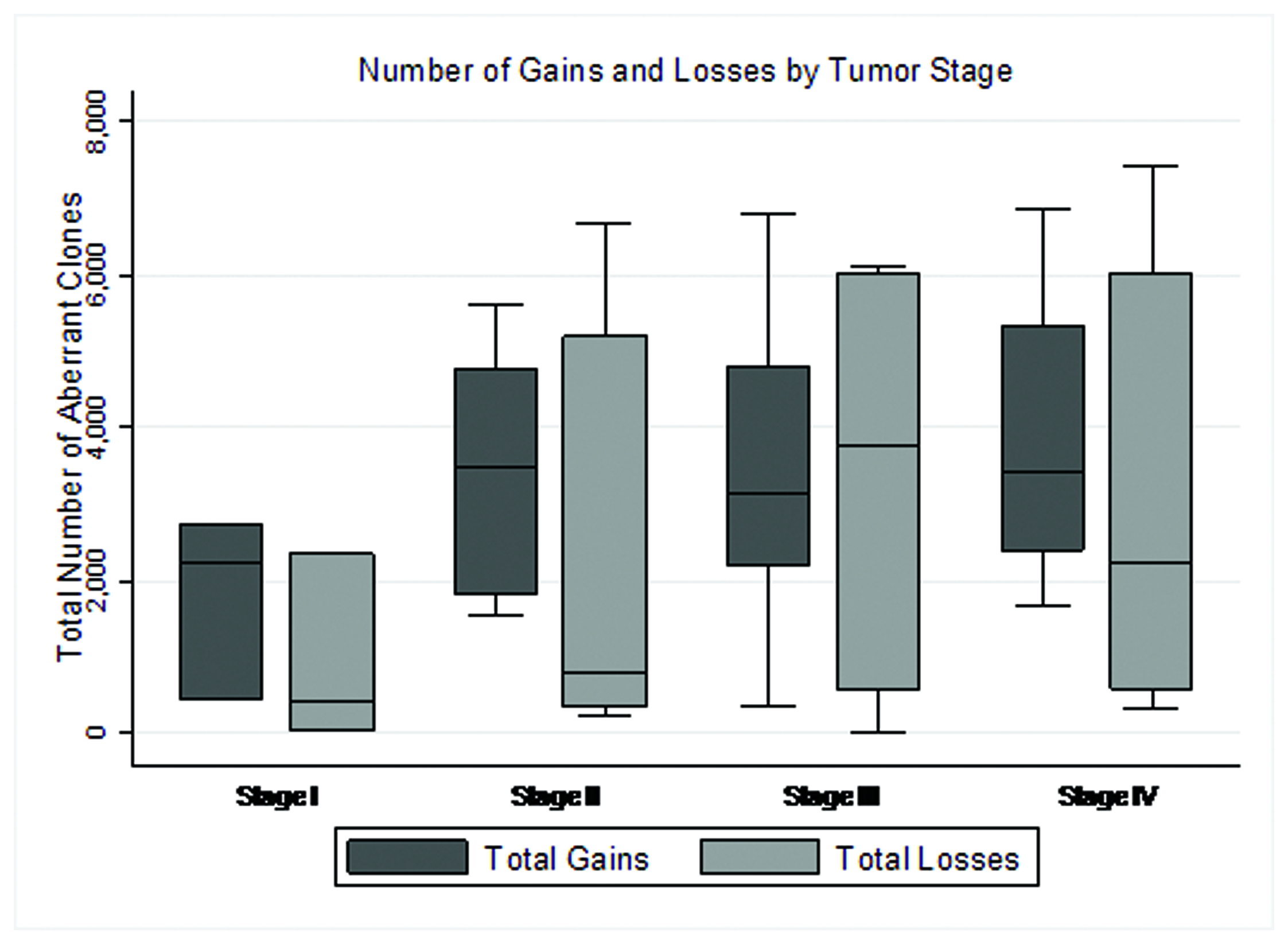

Supplement: Additional file 3 — Number of gains and losses by tumor stage. Boxplot of the number of gained and lost clones according to tumor stage. [file 1471-2407-8-98-S3.tiff]
